# Learning to be patient-centred healthcare professionals: how does it happen at university and on clinical placements? A multiple focus group study

**DOI:** 10.15694/mep.2020.000053.1

**Published:** 2020-03-26

**Authors:** Sheeba Rosewilliam, Vivek Indramohan, Richard Breakwell, John Skelton

**Affiliations:** 1 University of Birmingham; 2 Birmingham City University

**Keywords:** Clinical placement, Curriculum, Healthcare students, Patient-centred skills, Patient-centred attitudes

## Abstract

This article was migrated. The article was marked as recommended.

**Background**: Developing patient-centred skills in health professional students relies on their learning experiences at the university and on clinical placements. It is not known what students perceive about their teaching on patient-centredness and their views to develop the curriculum in this aspect.

**Methods**: Multiple focus groups were conducted with students who had experienced a minimum of two clinical placements from Medicine, Physiotherapy, Nursing and Speech and language therapy programs. Thematic analysis was conducted independently by two researchers and then themes were compared and integrated.

**Findings**: Five focus groups with 26 participants with a mean age of 23.8 years contributed to 286 minutes of recorded data. The key findings were that their curriculum focussing on patient-centred skills used artificial methods and teaching focussed largely on biomedical aspects, but, shared modules and specialist training enabled learning. Longer and diverse placements with good role models to emulate, enabled learning. As strategies they suggested reflections and role-modelling were vital along with further interprofessional working, goal-setting and understanding of human psychology.

**Conclusion**: Though the study is limited by its generalisability, strategies suggested by students can be further developed by superimposing them on learning theories. These strategies need to be tested in future studies.

## Introduction

Patient-centred care (PCC) is an individualised and relationship-centred, holistic approach that aims to empower patients ( Mead and Bower, 2000; Michie, Miles and Weinman, 2003). This approach is framed as a fundamental need to provide high quality care ( National Quality Board, 2013; Office of Patient Centered Care and Cultural Transformation, 2017). Unsafe practices and poor quality of care has been consistently attributed to a lack of adoption of a patient-centred care approach. It has been found that clinicians in the NHS adopt a task based approach instead of a patient-centred approach despite its benefits of positive patient-satisfaction and health outcomes ( Office of Patient Centered Care and Cultural Transformation, 2017).

In this climate, development of patient-centredness in health-professional students in different disciplines such as Medicine, Nursing and therapies could be supported through education and training. However, previous work has shown that medical education was too bio-medically focused ( Christianson *et al.*, 2007), students face pressures in practice and have low confidence ( Bombeke *et al.*, 2010) that were not conducive to adopt a PCC approach. Bombeke *et al.,* (2012) further identified that medical students struggled to transfer their learning of patient-centred skills to practice. There is limited information within literature on challenges faced by other health-professional students who work along with doctors in healthcare teams. These students form the future healthcare workforce; hence, it is important to understand how these students from different health professions are trained to deliver PCC.

Differences might arise in adoption of PCC behaviours amongst health-professional students due to the different approaches to undergraduate training such as the ‘caring model’ for nursing (Sowatsky *et al*., 2009, Beck, 2001), and ‘scientific model’ for medicine (Ronaghy, 2013). However, learning in the clinical context is influenced by the social and cultural context of learning based on social constructivist theories ( Torre *et al.*, 2006). Learning of patient-centred behaviours takes place in groups and is influenced by interaction with members (students, educators, patients, families) in multidisciplinary groups and through observing interpersonal and intrapersonal behaviours ( Braungart and Braungart, 2008) of other health professionals. Hence it is opportune to bring together students from various healthcare disciplines in a single study, to understand the influences on the development of PCC skills in health professional students.

Exploring students’ perception is essential for a teaching framework that is student-centred and could truly engage students in learning PCC skills ( Howe, 2001); yet, there is limited research around the perceptions of students about teaching and learning opportunities around developing PCC skills, their personal situation within the clinical learning context, and the culture of this context. Further, it is important to understand what students might recommend as supportive strategies to learn PCC skills. This knowledge is vital to develop a framework for a student-centred curriculum that can be adapted for different health professions since the philosophy of patient-centredness overarches all healthcare disciplines.

This study therefore aims to explore perceptions of students from four healthcare professions (Medicine, Nursing, Physiotherapy and Speech and Language Therapy) who form the majority of the healthcare workforce (NHS England, 2015), to understand their learning and teaching opportunities they had experienced and their recommendations regarding strategies to learn patient-centred care.

## Methods

The researchers were educators with a critical realist view who aimed to understand students’ perspectives around current teaching and learning within higher education to create awareness through description and transform practice ( McEvoy and Richards, 2003). A mixed-methods study that employed a cross-sectional parallel mixed design was planned (Teddlie and Tashakkori 2009, p. 151) with quantitative and qualitative components ( Rosewilliam *et al.*, 2019). A part of the qualitative data collected to explore the teaching of patient-centred care, learning opportunities and students’ own suggestions to deliver patient-centred strategies within curriculum, is reported here. We used focus groups to collect data as students can relate their experiences and reactions with their cohort due to their common context of learning ( Kidd and Parshall, 2000).

### Setting and sample

Three universities that delivered programmes for different health disciplines across the West Midlands in the UK were approached for participation. Two of the universities agreed to participate, while the third university refused based on the potential sensitivity of the data and overloading undergraduate students with research. The following undergraduate disciplines were approached to be in the study: medicine (MBChB), physiotherapy, nursing, and speech and language therapy (SALT). The sampling strategy was purposive in that the students should have had a minimum of two clinical placements since we wanted to capture well-informed and experienced students from the different health disciplines. We planned to get a sample size of 6-10 participants ( Gill *et al.*, 2008) from each group, however were bound by the ethical principal for non-coercion of students. For physiotherapy, nursing and SALT, students approached were in the third year of their studies in 2017, which was their final year as a student. In medicine, data was collected from fifth year students which was their final-year and also the third-year students - the latter on par with the students from other programmes sampled.

Students were given participant information sheets and consent forms to sign if they were willing to participate. The study had ethical approval from the Science Technology Engineering and Mathematics (STEM) ethics committee at the lead university (Ref. ERN_17-0413).

### Data collection method

The focus group discussions were held at a time convenient for each of the participating cohorts in a room within their respective university. The FG guide had been previously piloted with a group of physiotherapy students; and it had questions relevant to how students were taught about delivery of patient-centred care at university and on placements, and how this teaching could be improved to enhance their learning of patient-centred attributes. The focus groups were conducted by a researcher who did not teach on that programme, assisted by another researcher who took notes during the session. All researchers had master’s qualification and previous experience of conducting focus groups.

### Data analysis

The focus group discussions were digitally recorded and transcribed by an external agency. Data analysis followed the principles set out by Miles and Huberman for thematic analysis ( Miles and Huberman, 1999; Miles, Huberman and Saldana, 2014). Each researcher analysed two transcripts independently. The researchers analysed transcripts unrelated to the programmes they taught on, to minimise bias in their interpretation of data. Following coding of the first set of data, each researcher met with the second researcher to discuss their codes and themes. The researchers further clarified categories and developed their analysis based on the second coder’s analysis. A sample of this analysis is provided for audit trail (supplementary file 1). The first author was present for all the analysis meetings to have an overview of the interpretations of each analyst involved, and this helped to bring together themes and sub-themes from the various focus groups. The final integration and interpretations were additionally reviewed by all the researchers involved for greater confirmability.

## Results/Analysis

Five focus groups were held (one from nursing, physiotherapy, SALT and two from medicine year 3 and year 5), with 26 (four male and 22 female) participants in total. The number of placements for the various disciplines differed. Physiotherapy students had done 4-6 placements, third year medical students and SALT students had experienced 1-3 placements, nursing students had completed 7-10 placements and final year medical students had done more than ten placements by the time they participated in the study. Group sizes ranged from 3-7 students. In the third-year medical student group, participant numbers dropped to three since students who had consented, failed to attend the focus group. Age range was 20 - 42 years, with an average of 23.8 years. The focus groups lasted from 27 minutes to 93 minutes, an average of 57 minutes, with a total time of 286 minutes. Interestingly, the majority of the SALT students involved in this study had joined their programme as mature students. The participants’ demographics are presented on Table 1.

**Table 1.** Participants’ characteristics.

No.	Programme	Year of Study	Gender	Age
1	SALT	3	F	23
2	SALT	3	F	35
3	SALT	3	F	42
4	SALT	3	F	22
5	SALT	3	F	-
6	SALT	3	F	28
7	SALT	3	F	29
8	Physio	3	M	20
9	Physio	3	F	20
10	Physio	3	F	20
11	Physio	3	F	21
12	Physio	3	F	21
13	Physio	3	M	21
14	Physio	3	F	25
15	MBChB	3	F	21
16	MBChB	3	F	21
17	MBChB	3	F	20
18	MBChB	5	F	23
19	MBChB	5	M	23
20	MBChB	5	M	23
21	MBChB	5	F	22
22	MBChB	5	F	25
23	Nursing	3	F	28
24	Nursing	3	F	22
25	Nursing	3	F	21
26	Nursing	3	F	21

Themes and sub-themes:

The key themes and sub-themes have been presented in Table 2 along with key findings compared across disciplines. The themes are described below, with supporting quotes from each discipline. The year of study for the medical students is indicated as FG3 or FG5 for a medical student in year three or five beside the quote since this discipline had participants from two different year groups.

**Table 2.** Illustration of research themes and key constructs identified across disciplines.

Research Question	Key Themes	Key constructs	Perspectives of Medical students	Perspectives of Nursing students	Perspectives of Physiotherapy students	Perspectives of SALT students
Can you tell us how you are taught within your course about what is patient-centred care and how it can be applied in practice?	Biomedical, Fragmented and Artificial teaching methods at University. Current Strategies	Curriculum content in early years:	Modules largely focused on biomedical knowledge	Shared modules with other nursing streams improved perceptions.	Specific modules on PCC enabled understanding.	Theory of models of care helpful to relate to PCC.
		Case studies and simulations:	Case studies are unrealistic	Simulators are artificial		Simulation and case studies are useful for practice in a safe environment.
		Sessions on communication skills:	Skills taught by specialists from interactive unit.			
Can you tell us how you are taught on your clinical placements about what is patient-centred care and how it can be applied in practice?	Learning from placement experiences.	Length and diversity of placements:	Placements were short and less diverse which did not help improve awareness or continuity of care. Unscheduled time with patient helps Geographical location of placements exposed them to cultural diversity	Placements were longer and diverse which helped understand variability in PCC.	Placements were longer and diverse which helped understand variability in PCC.	Placements were shorter, fewer and less diverse
		Role models	Scarcity of positive role models to emulate.	Scarcity of positive role models to emulate.		Observation of experienced therapists helped to learn PCC behaviours.
		Interprofessional learning opportunities	Interprofessional observation	Multidisciplinary meetings Bedside-handovers	Multidisciplinary meetings	Multidisciplinary communication
		Family focus, poignant and personal experiential learning		Experiencing difficult situations. Family involvement in care of patient. Personal experiences of ill-health.	More time as a student to establish rapport.	Handling patients’ difficult queries
What do you think is the best way to teach principles and delivery of patient-centred care?	Suggested strategies for a patient-centred curriculum	Prepping for the real world	Understanding of human psychology	Team-working through inter-professional learning	Patient-centred goal setting and creating awareness about limitations	
		Reflective practice	Self-reflection and awareness of personality	Reflections on practice with peers Placement debrief days	Paper-based patients and mentor facilitated reflections	Paper-based reflections
		Role-modelling	Feedback and deeper conversations with mentors		Feedback and deeper conversations with mentors Shadowing other professionals	Feedback from mentors
		Placement modifications	Earlier and continuity of placements. PCC principles reiterated on placement including site specific strategies			
		Patients as teachers	Patient mentoring on placements	Patients delivering Classroom sessions		

### Theme 1: Biomedical, Fragmented and Artificial teaching methods

In most programmes, students were taught in specific modules about the principles and application of patient-centred care using formats, such as case discussions, and had sessions on communication skills and clinical reasoning skills within their classrooms. However the teaching was fragmented since it lacked follow up in other modules or on placement.

*“They (specialists) come in like a couple of times a year, usually, and just sort of teach you communication techniques, or get you to do like role play sessions, or you watch role play and then say how it could be different.. Yes, I guess that helps show you some of the different communication techniques you can use”.* Medical FG3.

*“The teaching that we get.. so we get a professional practice module.. so that teaches us about like patient-centred care and how we should treat patients.. and think that we haven’t had that actual experience, but we can think back to the classes that we had”.* SALT FG.

Speech and language therapy students mentioned benefitting from the use of simulation and case studies, which the nurses and doctors critiqued as being too artificial.

*“I think when you’re focussing on something physical in front of you (simulator) then you’re not necessarily going to think about holistic things that you can’t see. Like you say if it’s a dummy you’re not thinking about its family or whether it goes to church or not, are you?”* Nursing FG.

*“Most of our patient information exposure we had in first and second year was like through really funny case studies..where each one would have like a certain theme, and it would be really funny and over-dramatised..It would be over-the-top, wouldn’t it?”* Medical FG3.

Though PCC was embedded within university curricula using various methods within different modules, students suggested the focus was largely on biomedical knowledge in the first and second years especially in the medical curriculum.

*“We’ve been at medical school five or six years, but we’ve had to learn all of the basic science and, obviously, that’s really important for patient safety, so like when it’s exams we’re quite focused on being able to treat acute conditions and things like that”,* Medical FG5.

### Theme 2: Learning on placement to develop as patient-centred professionals

Students were appreciative of the theory and skills introduced within the university setting, as they could practice these in a safe environment, however, all the groups suggested that PCC skills are practical skills and placement is predominantly where they apply principles and develop as patient-centred practitioners.

#### a) Length and diversity of placements

The nursing and physiotherapy students reported longer (4-6 weeks) whereas medical and SALT students placements lasting 1-2 weeks (except on GP placements). Students suggested that longer placements gave them the continuity of care to extend a patient-centred approach. Physiotherapy and nursing students suggested, that a wider range of placements also helped them understand differences in how patient-centredness can vary in different situations e.g. in pediatrics consulting with parents was considered patient-centred whereas in palliative care patient-centredness was seen as being comforting. The medical students suggested that the time spent with patients was key, this was possible during evening hours when they were on on-call duties.

*“The only time that I thought that I was more able to be involved that sort of patient-centred care and decision making was during the GP placement, which is where we have five weeks where we’re on our own with the patient, and we can come up with our own management plan, and we have more time to discuss with the patient things about their life, and their likes, dislikes, their thoughts on things”.* Medical FG5.

For some students from less diverse counties, the geographical location of the university was mentioned as a factor that enhanced students’ opportunity to appreciate the differences in populations such as ethnicities, cultures and their needs.

*“I think we’re really lucky being in Birmingham, because there’s such a sort of cultural variety that, actually, it almost sort of - because everybody likes to be cared in different ways, and I’ve had lots of people want different things, it makes you ask people, ‘Actually, what are your wishes. How would you like this done? Is this okay for you?’ and I think that’s key”.* Medical FG5.

#### b) Role models and Interprofessional learning:

Whilst all groups of students suggested that observing good role models taught them how to communicate in a patient-centred manner, the scarcity of positive role models was highlighted by all students, but particularly by medical students. However medical students suggested that junior doctors gave them feedback, which they found helped improve their PCC skills in contrast to senior clinicians who were rushed.

*“It’s lots of learning by the example of the people that you’re observing and being taught by”.* Physiotherapy FG.

*“I mean, you see good examples and bad examples, but from what I’ve seen, like the more senior consultants tend to be the ones who are most in a rush, or don’t stop to let people ask questions and things, and I think that’s probably part of their job in terms of they’ve got the most pressure on them and things, but I also wonder whether it’s the further away they get from med school where they’ve had all of this training, like it becomes automatic to just carry on rather than stopping to think as much”.* Medical FG3.

*“Some of the teaching fellows that we’d have at hospital, they’d be really good role models, I think. They’ll take you to go and see a specific patient, but not because of like science, but because they had a really nice rapport with the patient,”* Medical FG3.

Perhaps having limited number of patient-centred role models prompted some medical students to attribute their learning to be from nurses and therapists who looked at wider aspects of care.

*“I think sometimes it’s not just your educator. It’s like the other members of staff, by listening to like other members of staff on the ward, it can also help you think about the patient’s needs without having to go and ask them as well”.* Physiotherapy FG.

*“we spent a couple of days with the nurses, and the nurses drilled it into us quite a lot”.* Medical FG3.

Hence working with other professionals was identified as a key factor by most of the other student groups, which enabled them to develop a holistic understanding of patients’ needs. Often, multidisciplinary meetings and presentation of case reports to other team members further gave students opportunity to gain a holistic perspective about the patients’ needs.

#### d) Family focused, poignant and personal experiential learning in nursing practice:

Nursing students had certain unique learning from reflections and their model of caring. Experiencing difficult situations, where patient care had been compromised and dealing with distressed patients were considered as opportunities to learn from, through reflections and feed forward. They also considered family involvement as key to understanding needs and providing holistic care for patients. Importantly, patients’ families’ positive feedback about nursing students’ patient-centred care encouraged the nurses in their learning about patient-centred care.

“I’ve learnt the most, perhaps, not when things have gone wrong but when I feel care has been compromised because haven’t looked at a patient or a family in a holistic way”.

*“75 per cent/80 per cent of my day is spent talking to the parents and supporting parents and having that experience in first year working, not even with mentally ill adults but distressed and upset and concerned adults.. I can’t explain how beneficial that’s been because.. it’s an integral part of the role and you can’t deliver the care to the child that you want without supporting the parents”* Nursing FG.

Students mentioned that they had learnt how to be more patient-centred from their own experiences of ill-health and illness experiences of family and friends.

*“Yes, ... like, after I’d had my operation and you’re doing all the things that we see patients doing and you’re thinking, ‘Why are you doing that?’, and then obviously it’s the anaesthetic drugs or something”.* Nursing FG.

### Theme 3: Strategies to consider for a patient-centred curriculum

Students suggested that development of patient-centred attributes can be dependent on a person’s personality and morality. Whether they had a natural flair for these attributes or not, students suggested that personality was a building block and education should help shape and develop these attributes. Hence, they recommended strategies for a structured learning approach described in this section.

*“I think so because even if you were morally that way inclined, you might not realise you’re doing it, and say if you aren’t that way inclined, as soon as you are taught about it you’re conscious and you make more of an effort to do that”.* Physiotherapy FG *.*

*“If we have more of a framework to stick to, it does help those who are maybe not so patient-centred naturally..* *but some people, it just doesn’t come naturally, but they can be shown how to be patient-centred in a way that isn’t necessarily the same as other things”.* Medical FG5.

#### a) Prepping for the real world:

In general, all students suggested that the university should prepare them adequately to be aware of the principles and application before they go on placement. Whilst the nursing and therapy students suggested that interprofessional learning provided that sense of support in a team, physiotherapy students focused on patient-centred goal-setting and creating awareness about limitations in the real world.

*“We were looking at case studies to do with medicine management in different contexts and it was really good to have the pharmacy students and their expertise so that we can ask them questions that we don’t necessarily know..* *Instead of trying to be everything, .. trying to be the little bit of the pharmacist and the physio and everyone else, you can just fall-back on the fundamentals of your nursing care and that very much is patient-centred,”.* Nursing FG.

*“Getting the students to understand, like contextually, why sometimes you can’t have that patient-centred care. I think one of the hardest things for me on placement was like - because one time I couldn’t see like two people because of time and I got frustrated”.* Physiotherapy FG.

Whilst the nursing and therapy students were focusing on the pragmatic aspects of practice, the medical students were suggesting a better understanding of human psychology and mental health issues before they went out on placement. They suggested a need to understand their own personality and behaviours to help them identify their own aptitude for being patient-centred.

*“There was a missing chunk. It isn’t what you see a patient for in hospital, everybody’s got a story, and everybody, to some extent, will have had an impact on, maybe, their feelings or the way they think about things, or their attitudes, and that’s not the same as mental health, but it’s almost a caveat of that”.* Medical FG3.

*“I think that’s probably what’s missing from the course, is actually thinking about, okay, what situations do I feel comfortable in, how would I naturally interact with people in different circumstances, and actually, for me, what’s important to remember in circumstances, .. and you need to have an understanding of yourself before you can really tailor yourself to work with someone else”.* Medical FG5.

Contrary to the self-reflection suggested by medical students, physiotherapy and nursing students suggested reflection in peer groups wherein small groups of students could reflect on patient-centred issues encountered in practice or on paper-based cases. Mentor facilitated reflections on specific patients on placements were also suggested. They wanted reflections to help consolidate learning from placement, aided by debrief days at university.

*“What I’ve learnt the most from, like over my three years is just getting it, whether the students like who are really best because there’s only 16 of us in our little cohort. We can sit and share experiences and everybody will have different points of view, just like your patients will have different points of view. So learning to appreciate each other’s view is massively like influential in your ability to relate to your patients”.* Nursing FG.

*“..once you’ve been on placement you’ve got like a safe space to reflect on what’s happened and maybe share the frustrations about when you couldn’t provide patient-centred care and like the barriers to it. Like a chance to discuss it. It can be comforting to know that other people are experiencing similar things and then maybe talk about ways of overcoming it or to take in to your next placement”.* Physiotherapy FG.

#### b) Role-modelling:

The key need for learning patient-centred attitudes and skills suggested by all groups of students was to have role models who could be observed and who could observe their performance on placements. Students suggested allocated time for feedback and deeper conversations with mentors would help develop their patient-centred skills. Mentoring was not restricted to the same disciplines, as students suggested mentoring from other professionals or observing them was helpful. Due to the negative role models they had observed, they questioned the patient-centred skill levels of the clinicians responsible for their training and wondered if there was a need to develop training strategies for trainers.

*“Also by observing the clinician and you are travelling in the car to a school, nursery or home and they would talk about what they were expecting to do in that session and they you would observe them and things would change because the client would want something different and just by observing how they adapted their plans helped me to give me the confidence it’s ok to move away from what I had planned”.* SALT FG.

*“I think I learn the most from the wards/nurses or whoever that have that ethos..”* Nursing FG *.*

*“One was a junior doctor who watched me take a history from - it was a paediatric rotation, and he watched me take a history from a girl who was suicidal. So that was quite useful feedback, because it wasn’t the easiest communication”.* Medical FG5.

#### c) Placement modifications:

Medical students suggested that general practice placements gave them the time and freedom to interact with patients independently, and they wished they had started these placements earlier in their first and second years. They suggested it would be good to follow up these patients for a year to learn how to integrate various aspects of patient-centredness into longer term care.

*“...in the third year, because we have to follow-up two patients in our GP practices, and we had an exam on them at the end of the year, but we have to follow them up and see how their care is going and just talk to them about how they were diagnosed and treated. I think that could be more useful to, maybe, incorporate into first and second year as well”.* FG Medical3 *.*

Students suggested that the principles of PCC learnt at university should be reiterated on placement. Further, strategies specific to that placement, how to communicate with the multidisciplinary members and non-verbal patient-centred skills should be taught in short bursts on placement. The university and placement partners need to agree on expectations of students’ behaviours.

*“Just like when we have like teaching at hospitals in our firms, just like five minutes just to say, ‘This is how you should do a consultation’, or in the GP, just five minutes at the beginning of your placement, like, ‘I tend to get the patient to sit here. I sit here’”* Medical FG5 *.*

#### d) Patients as teachers:

Use of real patients in the university setting was suggested as an effective method to practice patient-centred attitudes in a safe environment. Patients could guide them on communicating with lay people, attending to holistic needs, individualistic problem-solving and gauging patient-readiness to treatment. Whilst physiotherapists suggested bringing patients into the classroom medics preferred patient mentoring on placements.

*“I think it’d be maybe good, like especially in first year before you go on placement if people aren’t going to be attracted or learn a lot from lectures, maybe have a real-life patient coming in just talking about their story in hospital and their experience..”* Physiotherapy FG.

*“And you get a bit of mentoring and support from those patients..‘Oh, I’m really glad that I’m involved. I’m really glad to be helping you’, so that was really nice to have just a bit of support outside of - like still within* *the course, but not necessarily just from the doctors, from patients as well”.* Medical FG3.

## Discussion

This study explored the views of current healthcare students from various disciplines about how they learn to develop patient-centred attitudes and skills at university and on placements and their suggestions for strategies to teach patient-centred skills. To our knowledge this is the first study that has integrated the findings from different health professional groups to build a theoretical framework for a future curriculum; a curriculum that engages and benefits students’ development as patient-centred practitioners. Students suggested that though university teaching laid the foundation for building PCC attributes, clinical placements provided an invaluable opportunity for learning and application of these skills. They identified ‘role models’ as the key tool for supporting, guiding and developing these attributes. University teaching and placement teaching were isolated, and they suggested strategies to build the gap and reinforce learning from each sector.

A key focus within the university settings in this study was on communication skills training (CST) to improve patient-centred attitudes in medical students similar to wider practice (von Fragstein *et al.*, 2008). Though CST raised awareness about patient-centred communication, medical students found their skills were non-transferable when they faced pressures of time, resources, conflicts around their own learning needs on placements ( Bombeke *et al.*, 2012). One option is to embed CST within clinical training, rather than just within university, to diminish the impact of factors that limit transference of these skills.

Role modelling, a key element of Bandura’s social cognitive theory, came through as the vital element for learning patient-centred care. Placement educators are in an ideal situation to act as role models, provide opportunities and exposure to situations for developing patient-centred attitudes. For example, a clinical educator can ask a student to explore patient’s psycho-social needs in preparation for a ward round and communicate to the team during the actual rounds. However, with the increasing pressures in the workplace and the pre-existing work cultures ( Al-Bawardy *et al.*, 2009), it is necessary to train the trainer to develop patient-centred attributes ( Christianson *et al.*, 2007). Previous studies have shown that qualified clinicians showed improved tendencies to be patient-centred following a ten-hour training programme ( Dwamena *et al.*, 2012). Taking a step back, there is still a need to explore what clinical educators perceive of their responsibilities as a role model for students’ development as patient-centred practitioners.

Inter-professional learning (IPL) is gaining prominence as a method of teaching and learning to improve aspects of holistic understanding and cohesive working within health professional education ( Arenson *et al.*, 2015). Research suggests that IPL can improve patient perceived outcomes such as quality of care ( Hallin *et al.*, 2011) and satisfaction ( Reeves *et al.*, 2013). This study indicates that learning from professionals in other disciplines provides insights into holistic care, whilst inculcating caring attitudes from a different professional culture. Thus, shadowing of other professions on placements can be included as a strategy for IPL to overcome some of the organisational challenges that formal IPL poses ( Arenson *et al.*, 2015).

The social and cognitive learning theories highlight the personal situation, learning context and cultural influences as factors that need to be negotiated for individualistic response to learning. Students in this study have suggested that their own personality and morality, their personal experiences of illnesses and practice environments as influencing their learning of patient-centred attitudes. The challenge for the educators will be to untangle the complex interaction of these factors in teaching someone to be patient-centred. However, as students themselves suggest, broad frameworks can be used to develop these attitudes. Adult learners can adapt these frameworks to their learning needs ( Braungart and Braungart, 2008).

Limitations of this study can be identified as being restricted to one geographical region of the United Kingdom, and the limited sampling that can reduce the transferability of the findings. Recruitment could have been improved if a course tutor had approached the student cohorts, but this strategy was not employed for ethical reasons. However, since the context and participant characteristics have been laid out, readers can compare and extrapolate findings relevant to their practice. Data checking by individual participants was not possible as students left after completing their final year of studies. However, summary of discussion points was presented to each group at the end of each meeting to seek clarification ( Kidd and Parshall, 2000). Biases during interpretation were limited by involvement of multiple analysts and a postgraduate student (for a student perspective) to analyse and interpret part of the data ( Ruff, Alexander and McKie, 2005). Future research should look at perspectives of current role models, explore training of educators to plug the gap in evidence around role modelling and effects of educator training on development of patient-centred skills in students.

## Conclusion

In conclusion this study has explored learners’ perspectives about current local practices of teaching to be patient-centred and how these can be improved. The findings from the study reiterate from the students’ perspectives that they need role models and structured teaching of patient-centredness using frameworks, feedback and continuity in learning across sectors. Branch (2014) was optimistic in his prediction that modern education is creating humanistic medical professionals. There is still scope for developing ways to enable students to be patient-centred from knowledge that is derived from learners’ perspectives so that they engage with the student-centred curriculum.

## Take Home Messages

• University and clinical placements should share expectations about students’ behaviour.
• Patient-centred strategies should be set-out should be explicitly on placements.
• Students should be encouraged to reflect on patient encounters and emotions evoked during the experience.
• Educators should provide opportunities for students to demonstrate patient-centred behaviours.
• Assessments should be aligned with evaluating PCC attributes.

## Notes On Contributors

**Sheeba Rosewilliam** is a lecturer in the School of Sports, Exercise and Rehabilitation Sciences whose teaching of rehabilitation sciences is underpinned by the principles of patient-centred care. Her research focusses on rehabilitation interlinked with her pedagogical interest to improve training methods for the development of students’ skills to enable them to deliver patient-centred care.

**Vivek Indramohan**, a senior lecturer and programme director for the Biomedical Engineering programme is constantly adapting approaches to enhance student engagement and employability. Vivek was a principal Investigator in three pedagogic research projects to investigate the experience of students undertaking Bioscience / STEM curriculum and preparing for their future employability.

**Richard Breakwell** is a Senior Lecturer in the Department of Nursing. With a clear background in nurse education and theory, Richard is particularly interested in the pedagogy that underpins nurse education and curriculum development. Richard’s research focus is on student learning in both university and clinical practice.

**John Skelton**, Professor of Clinical Communication is the author of Language and Clinical Communication: this bright Babylon, which draws on his background in Applied Linguistics. He has published in excess of 100 articles on aspects of clinical communication, medical education, medical humanities, and applied linguistics including The Lancet.

## Declarations

The author has declared that there are no conflicts of interest.

## Ethics Statement

The study had ethical approval from the Science Technology Engineering and Mathematics (STEM) ethics committee at the lead university (Ref. ERN_17-0413).

## External Funding

School of Sports exercise and rehabilitation Sciences, University of Birmingham’s seed funding supported this project.
