# Supplementary material for: Learning to be patient-centred healthcare professionals: how does it happen at university and on clinical placements? A multiple focus group study
Source: MedEdPublish (2016). 2020 Mar 26;9:53. [Version 1] doi: 10.15694/mep.2020.000053.1 (PMC10697551; doi:10.15694/mep.2020.000053.1)
Supplement: Peer review history

# Reviewer report: Reviewer response for version 1

**DOI:** 10.21956/mep.20002.r30477

**Published:** 2020-04-12

**Authors:** Subha Ramani

**Affiliations:** 1 Harvard Medical School, Brigham and Women's Hospital

This review has been migrated. The reviewer awarded 5 stars out of 5 I comment the authors for conducting this study on patient-centred care and I agree that their learning experiences influence how they acquire these skills. I might venture to say that clinical placements more strongly impact increase in PCC skills than university based learning, as the hidden and informal curricula are more important than the formal curriculum. What they learn at the university as part of their formal curriculum can either be reinforced and enhanced or undone by what they learn in their clinical experiences.Given the caring model emphasized by nursing education, there is a lot to be said for interprofessional learning especially for medical students. If medical students are not engaged with other professionals, they might continue to assume that the science based or task based learning are more important- based on social constructivist theoretical principles.The problem statement is well written and real, study objective is clear and the mixed methods adopted are appropriate. The authors justify data collection methods and they purposively sampled a variety of health professional students. Ethical approval was obtained.The authors collected focus group data with rigour- after piloting, conducted by a researcher with no affiliation to the programme and observed by another researcher and both were educationalists. Data analysis is clearly described and cited. The authors describe their qualitative analysis step by step which will enhance dependability and transferability. The themes are very well described and quotes are representative of themes. I like the fact that one of the themes focussed on strategies to design PCC. The theme related to role-modelling is not surprising and it is good to see that students recognized its importance. The central importance of engaging patients as teachers is another critical theme.I like the emphasis on role-modelling and interprofessional learning in the discussion section, and agree with the limitations as noted. The authors have demonstrated rigour in several ways- triangulation, piloting, member checking etc.I enjoyed reading this well written paper very much as it deals with an important topic and outlines strategies for other health professions educators to enhance PCC very clearly. I would say that these findings and recommendations are important to all health professions educators whether they are involved in designing PCC or not. Regardless of their educational role, they are all role-models and need to be conscious about their communication skills with learners, other teachers, patients, families of patients and all healthcare professionals.

Reviewer Expertise:

NA

No decision status is available

# Reviewer report: Reviewer response for version 1

**DOI:** 10.21956/mep.20002.r30475

**Published:** 2020-04-08

**Authors:** Puja Dulloo

**Affiliations:** 1 Pramukhswami Medical College

This review has been migrated. The reviewer awarded 5 stars out of 5 Interesting article, since patient centered care is important component of the recent curriculum within our country. Every aspect of the article was clear and well explained. In methodology data collection and analysis was explained in detail. Majority of themes and sub-themes framed were interesting. I liked the theme “Role models & interprofessional learning” highlighting the scarcity of positive role models, particularly in medical schools. Working with other professionals enabled students to develop a holistic understanding of patients’ needs, which in my opinion, will allows students to communicate well with the patient thus have better patient care. This approach, probable, would create good repo among the student and patient. Discussion was well written, although a question, as already asked by one of the reviewer, was there difference of opinion as per the age of the students’ since there were young and middle aged students.Over all I enjoyed reading it.

Reviewer Expertise:

NA

No decision status is available

# Reviewer report: Reviewer response for version 1

**DOI:** 10.21956/mep.20002.r30474

**Published:** 2020-04-01

**Authors:** Tharin Phenwan

**Affiliations:** 1 University of Dundee

This review has been migrated. The reviewer awarded 4 stars out of 5 This article provided students’ perceptions of their learning on person-centred care and their recommendations from different disciplines.The background introduction part was clear, with relevant literature. The methods section was well-described. Readers can follow the authors’ process easily. DiscussionNot a comment but rather a question. There were several mature students in this study. Should they use the same framework, compared to younger students?Strengths/limitationsThe strengths and limitations were well described. I would not worry about the issue of generalisability since this is the qualitative study and the findings will be in-depth and relevant on that context with that participants and researchers. Data checking was also a good way to improve the rigour but the authors already have other methods in place so it was fine and robust.This article will be of interest to educators and healthcare professionals who are involved with interprofessional educations, curriculum developments, curriculum evaluation, and professionalism.

Reviewer Expertise:

NA

No decision status is available

# Reviewer report: Reviewer response for version 1

**DOI:** 10.21956/mep.20002.r30476

**Published:** 2020-03-31

**Authors:** P Ravi Shankar

**Affiliations:** 1 American International Medical University

This review has been migrated. The reviewer awarded 5 stars out of 5 This is an interesting and detailed study examining the teaching-learning of patient-centered care at a university in the UK. The authors have examined the perceptions of students of medicine, nursing, physiotherapy and speech and language therapy. The authors have provided a detailed description of the methodology of the study. The results section is comprehensive and detailed insights have been obtained both regarding the strengths of the program and how to further strengthen areas of weakness. The free-text comments are the strength of the study. I enjoyed reading them. The limitations have also been mentioned. The study will be of interest to most medical educators as patient-centered care is becoming an increasingly important component of the curriculum.

Reviewer Expertise:

NA

No decision status is available
